# High Dose Rate 192-Ir-Brachytherapy for Basal Cell Carcinoma of the Skin using a 3D Printed Surface Mold

**DOI:** 10.7759/cureus.4913

**Published:** 2019-06-17

**Authors:** Stephanie Casey, Gaurav Bahl, Joseph B Awotwi-Pratt

**Affiliations:** 1 Radiation Oncology, British Columbia Cancer Agency, Abbotsford, CAN; 2 Medical Physics, British Columbia Cancer Agency, Abbotsford, CAN

**Keywords:** 3d printed mould, surface mould, brachytherapy, skin cancer, squamous cell carcinoma, basal cell carcinoma, skin brachytherapy, 3d printing

## Abstract

We report on the treatment of a Basal Cell Carcinoma of the skin with high-dose-rate (HDR) brachytherapy using a 3D-printed surface mold. The lesion was treated with 40 Gy in 10 fractions, administered every second day. The treatment was well tolerated and there were no significant toxicities. The patient had a complete response to radiation therapy. So it can concluded that 3D printed surface molds can be effectively used in the context of HDR skin brachytherapy.

## Introduction

High-dose-rate (HDR) brachytherapy is a mode of treatment for superficial skin tumors that can arguably provide a more conformal or superficial dose distribution, and so potentially less toxicity and better cosmesis compared to external beam radiotherapy (EBRT) [1-5]. Three-dimensional (3D) printing is generating much interest in the technology for its potential applications in radiotherapy. This technology has much potential in improving brachytherapy use and radiation delivery in cases of irregularly-shaped anatomies, such as in the head and neck or lower limbs, where skin cancers are often present and critical structures are often located within close proximity. Some centers are moving towards the use of 3D printed molds in clinical practice for these reasons [6-8], and research has also been published on the feasibility of this [6-10].

This paper reports on our experience with the use of a brachytherapy surface mold created using 3D printing and used to treat a patient with basal cell carcinoma (BCC).

## Case presentation

**Figure 1:**
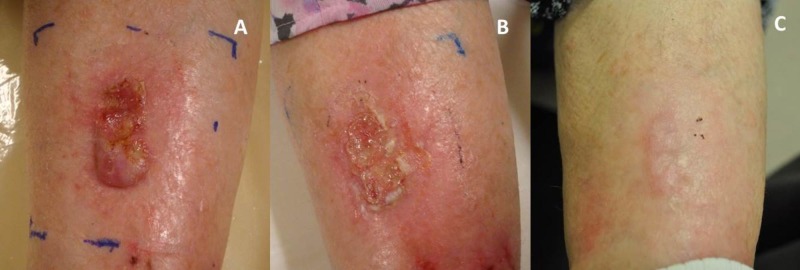
Lesion at the initiation of brachytherapy (A), at the end of brachytherapy (B), and at 12 weeks post-treatment (C)

An 82-year-old woman presented with a recurrent lesion on the skin of the left anterior shin. A biopsy had confirmed nodular BCC. Further surgery was not recommended due to the lesion’s characteristics (location, size) as well as the patient’s age and comorbidities. The clinical examination noted a 6 x 3 cm, well-circumscribed, raised lesion with significant scabbing, ulceration, and adjacent erythema. The lesion was not fixed to the bone. HDR brachytherapy was offered for definitive treatment. Informed consent was obtained for treatment and for this report. The scab was removed and the lesion was outlined to mark the gross tumor volume (GTV) (Figure 1A). A 1 cm expansion was added to create the clinical target volume (CTV) with no margin for planning target volume.

Typically at our center, an Aquaplast mold is created at the time of CT simulation and a Supraflab surface mold containing channels for brachytherapy catheters is placed directly on top. This generally allows for accurate setup, with close contact between the lesion to be treated and the surface mold. However, in this case, significant air gaps were noted between the Supraflab and Aquaplast on the CT images. While some could be attributed to the curvature and angles of the limb, the irregular height of the lesion also played a role. The decision was then made to use a 3D-printed surface mold placed directly over the lesion to be treated. The goal was to assess whether a mold of more uniform thickness could be created with the 3D printer, with the uniform thickness between the catheter channels despite the irregularity in the treatment surface from the tumor and the curvature of the leg. We were interested to see whether the 3D printed material would also allow for better adherence to the skin surface. When the patient was re-scanned, a superior fit was found in terms of setup variability and air gaps.

The 3D printed mold was created using a ProJet MJP 2500 Plus 3D printer (3D Systems, Rock Hill, SC, USA). The material used to create the mold is a UV curable elastomeric material called VisiJet M2 ENT (3D Systems, Rock Hill, SC, USA). The 3D printed mold is tissues equivalent, with a similar density compared with our standard molds (1.21 versus 1.25 g/cm^3^, respectively). The mold was printed in 2.5 hours and measured 8 x 8 cm with a 1 cm total thickness (5 mm under all the channels).

**Figure 2:**
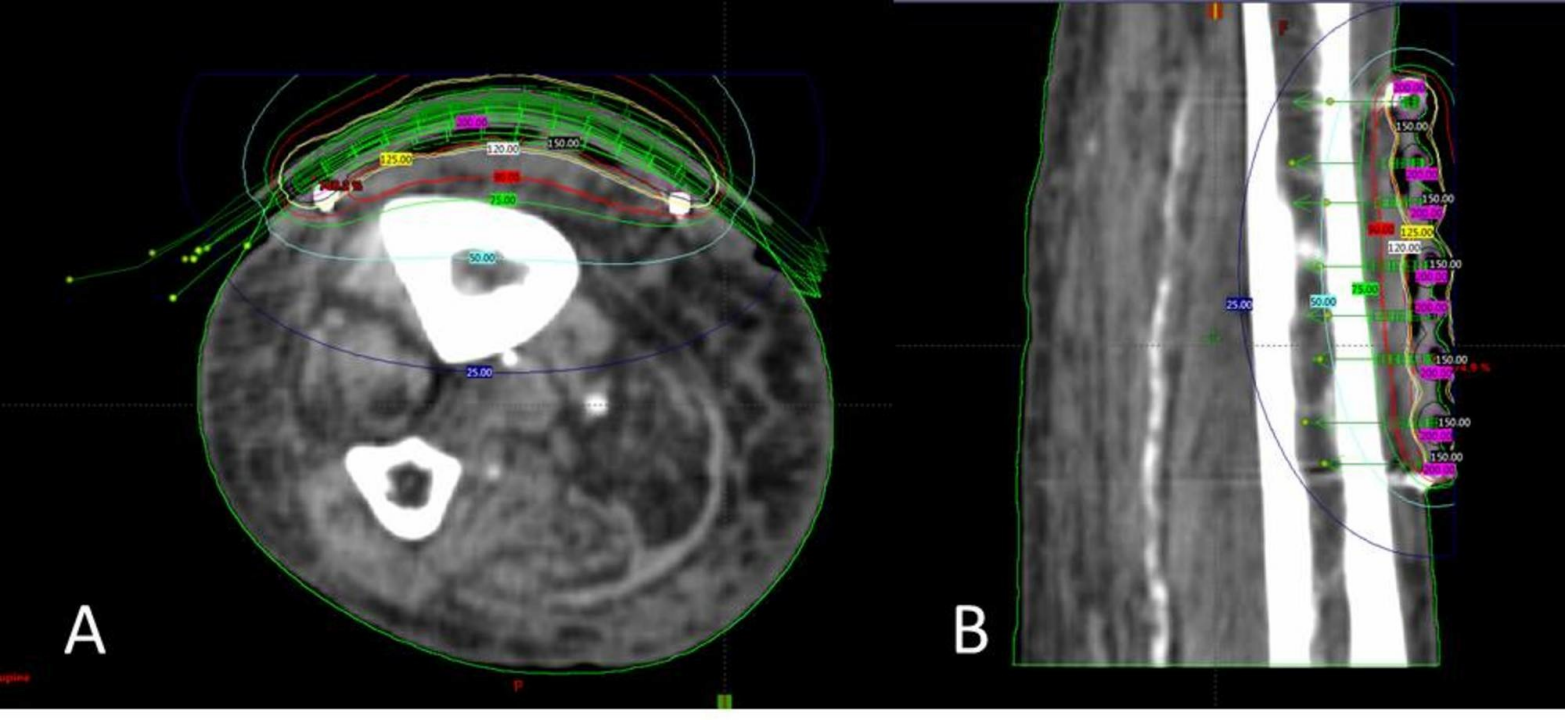
Axial (A) and sagittal (B) screen captures of the plan.

Brachytherapy treatment planning was done using the BrachyVision software (Varian Medical System, Palo Alto, CA, USA) (Figure 2), which assumes homogeneity of the calculating medium and does not account for air gaps. We prescribed 40 Gy in 10 fractions at a depth of 5 mm from the skin surface, using 8 catheters. Treatment was delivered using an Ir-192 source, on alternate working days. Our institutional dosimetric objective is to cover the CTV with the 90% isodose line. For this patient, the target volume was calculated as 10.7 cm^3^, and 95.7% of the target volume was covered by the 90% isodose, the minimum dose (0.1 cm^3^) was 86.9%, and the median dose was 103.6%. The maximum skin surface dose was 127.9%.

Cosmetic and toxicity results were graded according to an RTOG/EORTC standardized cosmesis scale and CTCAE v.4.03, respectively, based on clinical notes and photographs taken during and after brachytherapy [11-12]. During radiotherapy, the patient had increased discharge at the site of ulceration (requiring regular dressing changes), erythema, scabbing and patchy moist desquamation (Figure 1B). These effects were classified as Grade 2 toxicities. At 12 weeks post-brachytherapy, the lesion had completely resolved (Figure 1C). The treated skin was mildly pink with no ulceration, discharge, or skin atrophy. The patient was happy with the cosmesis and outcome.

## Discussion

Many elderly patients prefer radiation therapy over surgery for treating skin malignancies due to the less aggressive nature of this modality. Brachytherapy can arguably provide more conformal treatment compared to EBRT and potentially result in less tissue being impacted by the dose delivered. If a smaller volume is treated, this has the advantage of leaving more options available should another lesion arise near the previously treated volume (EBRT volumes are generally larger, and can complicate subsequent treatments due to beam overlap). However, brachytherapy does require more meticulous planning, and surface molds, or implants, to stabilize the catheters and conform to the body contour.

**Figure 3:**
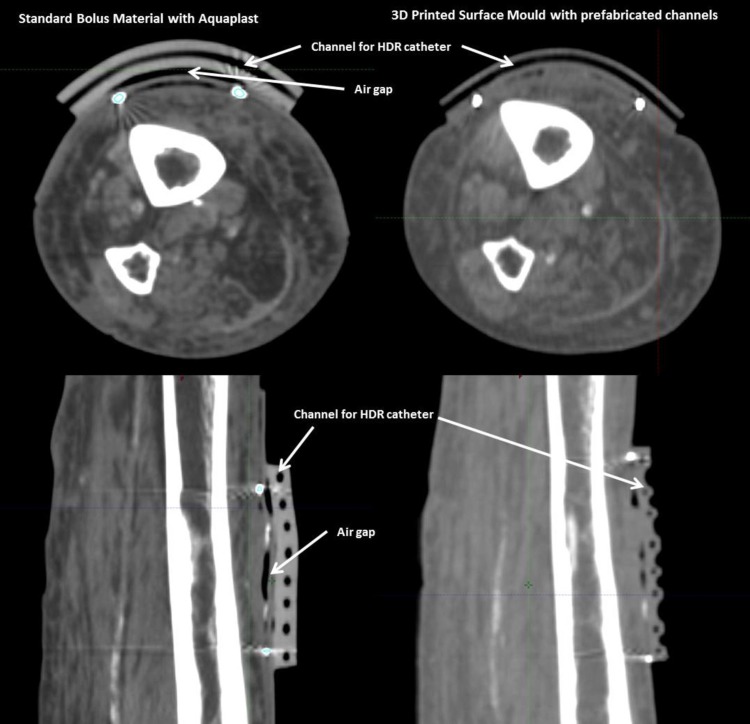
Axial and sagittal computed tomography images with standard vs. 3D printed bolus

In this case, the 3D printed mold was found to be superior to a hand-fabricated mold as it provided a much better fit to the particular contours of the patient and the lesion itself, with lesser air gaps (as demonstrated in Figure 3) and therefore a better plan in terms of coverage. Other potential advantages noted by our institution with the use of 3D printed molds - as opposed to hand-fabricated molds - include greater efficiency (the mold was more likely to fit well on the first attempt), time needed to create the mold (often it can be printed overnight), the generally better accuracy in terms of catheter placement and uniformity of mold thickness and finally, that it does not require the same level of expertise and materials to generate a mold as the one made by hand. That said, complex molds based on acquired CT images of a patient may require multiple versions and revisions, as there are sometimes unexpected changes or errors in the process of designing and printing the mold. 3D printers are not immune to errors but these are much easier to rectify, based on our experience so far. In general, there are also limitations as to the movement of the source. The source is capable of traveling in a complete loop (such as treating the entire circumference of a wrist) but is not capable of traveling through highly acute angles, whether in a 3D printed mold or hand-fabricated one. In our practice, an initial pre-treatment quality assurance check on the feasibility of the mold curvature, the angles the source wire has to traverse, and the treatment plan generated is paramount to ensuring successful treatment.

Overall, the bolus performed well and treatment proceeded without any issues. There was an expected level of toxicity similar to that seen in other brachytherapy and EBRT plans. At the 12 weeks follow up, there was no clinical evidence of the tumor, acute toxicities had resolved, cosmesis was good, and the patient was happy with the treatment.

There are obviously some limitations: this report details our experience with a single patient, and while we have some data related to short-term follow up, we have no long-term data.

## Conclusions

This experience provides confidence in using 3D-printed molds and the next step would be specifically generated molds based on individual patient contours. More research and experience will be needed in this regard, but it promises to be a very interesting technique.
